# Targeted Ultrasound With Contrast Enhancement for Reassessment of MRI‐Detected BI‐RADS 4 Nonmass Enhancement: Impact on Biopsy Reduction and DCIS Detection

**DOI:** 10.1155/tbj/1932231

**Published:** 2026-11-05

**Authors:** Qinghua Niu, Ting Sun, Mingxuan Li, Lianfang Du, Yingyu Cai, Lifang Jin, Chao Jia, Hui Li, An Chen, Fan Li

**Affiliations:** ^1^ Department of Ultrasound Shanghai Chest Hospital Shanghai Jiao Tong University School of Medicine Shanghai China shsmu.edu.cn; ^2^ Department of Ultrasound Qingdao Hospital University of Health and Rehabilitation Sciences (Qingdao Municipal Hospital) Qingdao China; ^3^ Department of Ultrasound Shanghai General Hospital Shanghai Jiao Tong University School of Medicine Shanghai China shsmu.edu.cn; ^4^ Department of Ultrasound Shanghai YangZhi Rehabilitation Hospital (Shanghai Sunshine Rehabilitation Center) Tongji University School of Medicine Shanghai China tongji.edu.cn; ^5^ Department of Ultrasound Huashan Hospital Fudan University Shanghai China fudan.edu.cn; ^6^ Department of Radiology Shanghai General Hospital Shanghai Jiao Tong University School of Medicine Shanghai China shsmu.edu.cn

**Keywords:** BI-RADS 4, biopsy reduction, breast MRI, contrast-enhanced ultrasound, DCIS, nonmass enhancement

## Abstract

**Objective:**

To evaluate whether targeted ultrasound (TUS) combined with contrast‐enhanced ultrasound (CEUS) can safely reclassify MRI‐detected BI‐RADS 4 nonmass enhancement (NME) lesions to reduce unnecessary biopsies while maintaining high diagnostic sensitivity for breast cancer.

**Materials and Methods:**

In this prospective study (2017–2023), we enrolled 169 MRI‐detected BI‐RADS 4 NME lesions in 167 women (23–84 years). All patients underwent TUS and CEUS within one week after MRI, followed by surgical pathology. We evaluated whether TUS combined with CEUS could reclassify lesions to avoid biopsy without compromising cancer detection.

**Results:**

Among 169 lesions (83 benign, 86 malignant), TUS detected 97.0%. Using predefined CEUS benign criteria, 36/83 benign lesions (43.4%) and 39/169 overall (23.1%) could have safely avoided biopsy. Diagnostic sensitivity remained high (96.5%, 83/86). CEUS significantly improved detection of DCIS, upgrading 87.5% (21/24) of MRI‐BI‐RADS 4a DCIS/DCIS with microinvasion to BI‐RADS ≥4b. False‐negative cases were limited to three lesions in challenging anatomical locations (subareolar or occult). Biopsy reduction was highest in asymptomatic patients, lesions ≤ 2 cm, homogeneous enhancement, and MRI BI‐RADS 4a.

**Conclusions:**

TUS combined with CEUS can substantially reduce unnecessary biopsies for MRI‐detected BI‐RADS 4 NME while maintaining excellent sensitivity. This approach is particularly effective for MRI BI‐RADS 4a lesions and small homogeneous NME and may provide a clinically practical strategy for improving diagnostic efficiency in breast cancer care.

## 1. Introduction

Dynamic contrast‐enhanced (DCE) MRI has become an important adjunct in breast cancer detection, particularly for high‐risk screening and preoperative staging [1, 2]. However, nonmass enhancement (NME) remains one of the most challenging MRI findings, with wide variability in morphology and enhancement kinetics that often lead to diagnostic overestimation [3]. BI‐RADS 4 NME, especially Category 4a, carries a low and inconsistent positive predictive value, resulting in a substantial number of benign biopsies and unnecessary procedures for patients [4, 5].

Targeted ultrasound (TUS) is frequently used to correlate MRI‐detected lesions, but NME typically lacks discrete sonographic morphology, limiting its ability to refine risk [6]. Contrast‐enhanced ultrasound (CEUS) offers additional microvascular information and may help differentiate benign from malignant NME, yet its clinical utility in this specific context remains inadequately studied [7]. Evidence regarding whether CEUS can safely reclassify MRI‐detected NME and reduce biopsy burden is limited, and no prospective data focused specifically on BI‐RADS 4 NME are available [8].

Given the high clinical cost of MRI‐driven false positives and the need for more precise risk stratification tools, establishing whether TUS combined with CEUS can reliably identify low‐risk NME and detect clinically significant lesions such as ductal carcinoma in situ (DCIS) is of substantial importance. This prospective study evaluates the performance of TUS and CEUS for reassessment of MRI‐detected BI‐RADS 4 NME, with the goals of reducing unnecessary biopsies while maintaining high sensitivity for malignancy.

## 2. Materials and Methods

### 2.1. Patients

In this prospective study, clinical and imaging data were consecutively collected from patients with breast lesions treated at a single tertiary‐care center between January 2017 and December 2023. The inclusion criteria were as follows: (1) patients with breast lesions detected on DCE‐MRI and classified as BI‐RADS Category 4 NME by consensus between two senior radiologists in accordance with the 5th edition of the ACR BI‐RADS guidelines [9]; (2) availability of complete and high‐quality TUS and CEUS imaging corresponding to the MRI‐detected lesions; (3) definitive pathological diagnoses obtained through surgical resection; (4) no prior biopsy or neoadjuvant therapy before undergoing DCE‐MRI or US examination; (5) no contraindications to CEUS, with all patients providing written informed consent prior to the CEUS procedure; (6) the interval between MRI and US examinations did not exceed one week; and (7) all imaging and surgical procedures completed within one month. This study was approved by the institutional ethics committee of a tertiary‐care hospital (approval no. 2023‐383). Patients who did not meet the inclusion criteria were excluded.

Of 9679 patients undergoing breast DCE‐MRI, 1916 were classified as BI‐RADS Category 4. Among 323 NME cases assessed with TUS and CEUS, 167 patients with 169 lesions were included after exclusions for lost MRI images (*n* = 95), incomplete pathology (*n* = 36), poor sonographic quality (*n* = 22), and metastatic lesions (*n* = 1) (Figure 1). The cohort comprised 83 benign and 86 malignant lesions, with mean ages of 45.3 ± 12.6 and 57.6 ± 11.3 years, respectively (*p* < 0.001).

**FIGURE 1:**
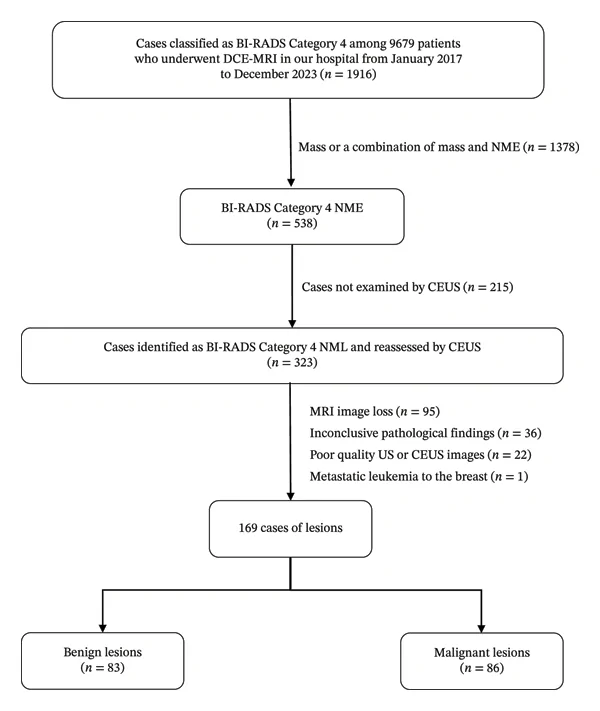
Study inclusion flowchart. Note. DCE‐MRI, dynamic contrast‐enhanced magnetic resonance imaging. BI‐RADS, Breast Imaging Reporting and Data System. NME, nonmass enhancement. NML, nonmass lesion. CEUS, contrast‐enhanced ultrasound. US, ultrasound.

### 2.2. MRI Examination

Breast MRI was performed using a 3.0T scanner (Philips Ingenia, Philips Healthcare, the Netherlands) equipped with a 16‐channel breast coil. The imaging protocol included axial T1‐weighted imaging with TR/TE = 609/8 ms, 36 slices, field of view (FOV) = 280 × 340 mm, matrix = 352 × 424, and slice thickness = 3.85 mm, followed by axial T2‐weighted imaging with TR/TE = 4000/70 ms, 36 slices, FOV = 280 × 340 mm, matrix = 280 × 318, slice thickness = 3.85 mm, and flip angle = 90°. Diffusion‐weighted imaging (DWI) was performed with TR/TE = 5098/70 ms, b‐values = 0 and 1000 s/mm^2^, 32 slices, FOV = 350 × 240 mm, matrix = 140 × 93, and slice thickness = 5 mm. Diffusion kurtosis imaging (DKI) was conducted with TR/TE = 4034/102 ms, b‐values = 0–3000 s/mm^2^, 32 slices, FOV = 350 × 240 mm, matrix = 140 × 94, and slice thickness = 5 mm. DCE T1‐weighted imaging was performed after intravenous injection of 0.2 mmol/kg gadolinium‐based contrast agent (Gd‐DTPA, Magnevist, Bayer Healthcare) at a rate of 3 mL/s, followed by a 20 mL saline flush. This sequence captured 29 phases at 9.7 s per phase, with TR/TE = 3.2/1.55 ms, FOV = 360 × 360 mm, matrix = 276 × 276, flip angle = 10°, and slice thickness = 3.0 mm.

### 2.3. Diagnostic Workflow for BI‐RADS Category 4 NME Lesions Using TUS and CEUS

Following the identification of BI‐RADS Category 4 NME lesions on DCE‐MRI, a TUS examination is conducted within one week. Two experienced radiologists (F.L. and C.J.) perform the examination using Aplio 500 and Aplio i900 systems (Canon, Japan), equipped with 14‐5L and 14‐8L transducers, respectively.

For localization of MRI‐detected lesions via TUS, the distance from the lesion margin to the nipple is considered the primary reference. Telegrafo et al. [10] reported this distance as a stable and reliable metric, with a minimal discrepancy of 0.7 ± 0.2 cm between MRI and TUS measurements. While the original validation by Telegrafo et al. was conducted primarily in a cohort of mass‐type lesions, subsequent studies applying a comparable distance‐ and landmark‐based localization strategy specifically to nonmass enhancement lesions have supported the applicability of this general principle to NME, albeit with lower detection rates than for mass lesions (MLs), reflecting the greater positional displacement and less distinct margins characteristic of NME [11]. Additionally, the lesion’s position relative to surrounding anatomical structures observed on MRI—such as breast glandular patterns, Cooper’s ligaments, fat morphology, vascular distribution, and adjacent abnormalities—facilitates precise localization during TUS. When a suspicious lesion is detected via TUS, it is classified as a ML or nonmass lesion (NML), with detailed ultrasonographic features documented according to the 5^th^ and 6^th^ editions of the BI‐RADS guidelines [9, 12]. Color Doppler flow imaging (CDFI) is then used to evaluate blood flow signals, with the color scale reduced to below 8 cm/s to detect small and low‐speed flow, and wall filtering minimized to prevent false positives.

After lesion confirmation on TUS, radiologists conduct CEUS at a low mechanical index of 0.07, with uniform overall and background gain set at 73 dB. The contrast agent SonoVue (Bracco, Italy) is prepared according to the manufacturer’s instructions, with 5 mL of contrast suspension injected intravenously, followed by a 5 mL saline flush. Timing starts concurrently with injection, and dual‐imaging is employed to prevent lesion displacement during contrast imaging. The probe remains stationary for the first minute, after which dynamic scanning of the suspicious area is performed to detect additional lesions.

### 2.4. CEUS Feature Assessment and BI‐RADS Classification Adjustment Criteria

The CEUS‐based downgrade and upgrade criteria described below were prespecified prior to the initiation of patient enrollment in 2017, based on the breast CEUS literature available at that time and the research team’s preliminary institutional experience, and remained unchanged throughout the study period [13, 14]. Image interpretation and BI‐RADS reassignment were performed at the time of each examination, with radiologists blinded to clinical and pathological information and prior to the availability of the pathological reference standard.

CEUS interpretation evaluates the following parameters: (1) enhancement time, (2) peak enhancement intensity, (3) enhancement morphology, (4) enhancement margins, (5) enhancement order, (6) contrast agent distribution, (7) presence of increased perilesional vascularity, (8) perfusion defects, (9) enhanced scope, and (10) contrast agent retention. In this study, three radiologists were involved in image interpretation. LF.J and YY.C independently interpreted the images. Discrepancies in interpretation were resolved through consensus among all three radiologists (LF.J, YY.C, and LF.D). The radiologists were blinded to the clinical and pathological data during the image review process.

For ML, benign features suggesting low malignancy risk on CEUS include synchronous or iso‐/hypo‐enhancement compared to adjacent breast glandular tissues in the arterial phase on CEUS or rapid, intense enhancement with circumscribed margins, regular morphology, homogeneous enhancement, no increased perilesional vascularity, and absence of contrast retention. Malignant features, indicating higher malignancy risk, include arterial‐phase hyper‐enhancement with irregular morphology, noncircumscribed margins, heterogeneous enhancement, increased perilesional vascularity, enlarged enhancement scope, and contrast retention. These criteria are consistent with enhancement principles reported in earlier breast CEUS studies [13, 14].

For NME lesions, benign features include iso‐/hypo‐enhancement relative to surrounding breast glandular tissues in the arterial phase on CEUS, consistent with general principles of breast CEUS enhancement kinetics established prior to study initiation; these features are also consistent with subsequently published NME‐specific data [15]. Malignant features, suggesting elevated malignancy risk, include intense arterial‐phase hyper‐enhancement with increased perilesional vascularity, contrast retention, and perfusion defects. Lesions primarily showing benign features are downgraded to BI‐RADS Category 3 or below, indicating low malignancy likelihood and supporting follow‐up rather than immediate intervention. Conversely, lesions predominantly exhibiting malignant features are upgraded to a higher CEUS BI‐RADS category (e.g., 4 or above), necessitating further diagnostic or therapeutic measures. The overall study design, including prestudy criteria development and the prospective per‐patient diagnostic workflow, is summarized in Figure S1.

### 2.5. Lesion Localization for Surgical Excision

On the day of surgery, the same radiologists who performed the original TUS/CEUS examination re‐localized each target lesion using conventional ultrasound, based on the previously recorded distance to the nipple and surrounding anatomical landmarks. The corresponding skin projection was marked directly on the patient’s body surface, and this marking, together with the documented imaging description of lesion depth and relationship to adjacent structures, guided the surgeon in defining the excision margins. This procedure, consistently performed by the same radiologist team throughout the study period, ensured correspondence between the lesion identified on MRI, evaluated by TUS/CEUS, and submitted for pathological examination.

### 2.6. Pathological Findings

All 169 lesions underwent surgical excision, in accordance with the study inclusion criteria requiring definitive pathological diagnosis via surgical resection, with the resulting histological diagnosis serving as the reference standard for all diagnostic performance analyses. We documented the pathological diameter of malignant lesions, grade, hormone receptor status, Ki‐67, and the status of human epidermal growth factor receptor 2 (HER2) [16, 17].

### 2.7. Statistical Analysis

Data processing was conducted using Python 3.8.19 (Python Software Foundation, USA). For quantitative data that conform to a normal distribution, results are expressed as the mean ± standard deviation; if not conforming to a normal distribution, they are expressed as the median (interquartile range). Qualitative data are represented as frequencies and percentages (*n*, %). To compare differences between groups, quantitative data were analyzed using the independent samples *t*‐test (for normal distribution) or the Mann–Whitney *U* test (for non‐normal distribution), while qualitative data were analyzed using the chi‐squared test or McNemar’s test. Correlation analysis was performed using Spearman’s rank correlation coefficient. Nonparametric bootstrap resampling was employed to estimate the 95% confidence intervals (CIs). A *p* value of less than 0.05 was considered the threshold for statistical significance. The consistency of image interpretation between TUS, CEUS, and DCE‐MRI was assessed using Cohen’s kappa coefficient.

## 3. Results

### 3.1. Histopathologic Characteristics

Malignant lesions included 50 DCIS, 27 invasive ductal carcinoma (IDC), 4 solid papillary carcinoma (SPC), and 5 others. Benign lesions included 28 adenosis, 27 inflammatory changes, 9 intraductal papillomas, 8 usual ductal hyperplasia, 7 atypical ductal hyperplasia, and 4 others. The proportion of BI‐RADS 4a was higher in benign (72.3%) than malignant cases (47.7%, *p* = 0.002) (Table 1). TUS detected 97.0% (164/169) of lesions (96.4% benign, 97.7% malignant). The kappa value for inter‐reader agreement was 0.810 (Table S1).

**TABLE 1:** Baseline demographics and clinical features of patients and lesions.

Characteristics	Benign lesions (*n* = 83) (%)	Malignant lesions (*n* = 86) (%)	*p* value
Age	45.3 ± 12.6	57.6 ± 11.3	<0.001
Menstrual state			<0.001
Premenopause	58 (69.9)	27 (31.4)	
Postmenopause	25 (30.1)	59 (68.6)	
Ipsilateral symptoms			0.025
Asymptomatic	30 (36.1)	28 (32.5)	
Palpable mass	32 (38.6)	36 (41.9)	
Nipple discharge	9 (10.8)	19 (22.1)	
Pain	12 (14.5)	3 (3.5)	
Location			0.699
Right	43 (51.8)	42 (48.8)	
Left	40 (48.2)	44 (51.2)	
Pathological type			
Benign			
Adenosis	28 (33.7)	—	
Inflammation	27 (32.5)	—	
IDP/UDH/ADH/others^∗^	9/8/7/4 (10.8/9.6/8.4/4.8)	—	
Malignant			
DCIS	—	27 (31.4)	
DCIS with microinvasion/invasion	—	3/20 (3.4/23.4)	
IDC	—	27 (31.4)	
SPC/others^#^	—	4/5 (4.6/5.8)	
DCE‐MRI BI‐RADS			0.002
4a	60 (72.3)	41 (47.7)	
4b	17 (20.5)	24 (27.9)	
4c	6 (7.2)	21 (24.4)	

*Note:* Others^∗^, including 3 fibroadenomas and 1 postoperative change. Others^#^, including 2 ILC with LCIS, 1 SPC with invasion, 1 invasive micropapillary carcinoma, and 1 mucous cancer with DCIS. IDP, intraductal papilloma.

Abbreviations: ADH, atypical ductal hyperplasia; DCE‐MRI, dynamic contrast‐enhanced magnetic resonance imaging; DCIS, ductal carcinoma in situ; IDC, invasive ductal carcinoma; ILC, invasive lobular carcinoma; LCIS, lobular carcinoma in situ; SPC, solid papillary carcinoma; UDH, usual ductal hyperplasia.

### 3.2. Correlation of TUS/CEUS Features With Pathology in BI‐RADS 4 NME Lesions

TUS identified 36 MLs (15 benign and 21 malignant) and 128 NMLs (65 benign and 63 malignant). Lesions were classified as BI‐RADS 3 (3/164) or 4 (161/164), with significant correlation to pathology (*p* = 0.049, *p* < 0.001). For ML on CEUS, pathological correlation was observed with enhancement time, peak intensity, enhanced shape, margins, contrast distribution, perfusion defects, perilesional vascularity, enhancement scope, contrast retention, and CEUS BI‐RADS (*r* = 0.357–0.832). For NML, correlated features included peak intensity, enhancement order, contrast distribution, perilesional vascularity, enhancement scope, contrast retention, and CEUS BI‐RADS (*r* = 0.271–0.652) (Figure 2). Inter‐reader agreement kappa values ranged from 0.822 to 0.957 (Table S1).

FIGURE 2Correlation analysis of ultrasonographic characteristics and pathological results of DCE‐MRI BI‐RADS Category 4 NME lesion on US and CEUS. Note. NML, nonmass lesion. ML, mass lesion. DCE‐MRI, dynamic contrast‐enhanced magnetic resonance imaging. BI‐RADS, Breast Imaging Reporting and Data System. NME, nonmass enhancement. CEUS, contrast‐enhanced ultrasound. US, ultrasound.

**Figure:**
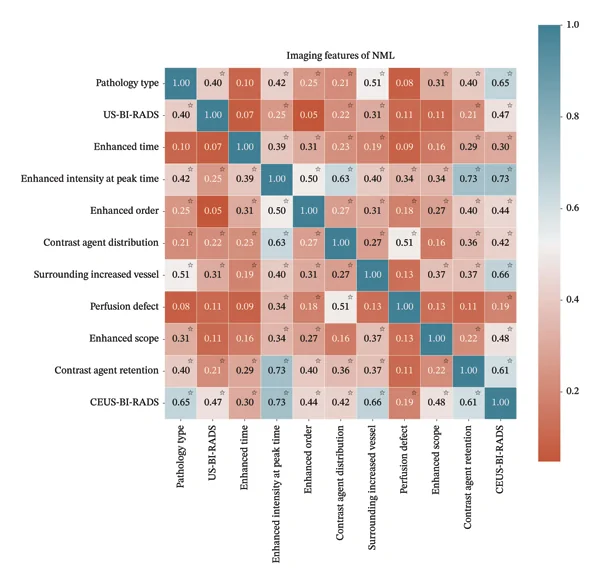

**Figure:**
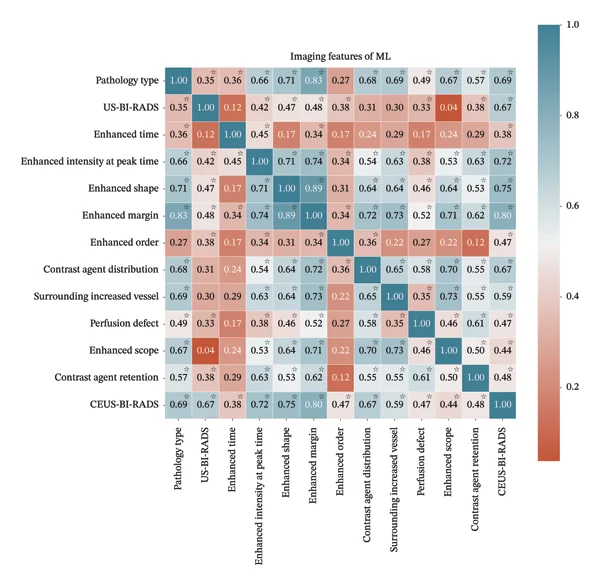

### 3.3. Diagnostic Performance of CEUS in Downgrading BI‐RADS 4 NME Lesions

TUS alone reduced the rate of biopsies for benign lesions by 7.2% and the overall biopsy rate by 4.7%, with a sensitivity of 97.7%. When TUS was combined with CEUS benign indicators, the reduction in biopsies for benign lesions was 43.4% (36 out of 83, 95% CI: 32.5%–54.2%), and the overall biopsy rate decreased by 23.1% (39 out of 169, 95% CI: 16.6%–29.6%), maintaining a sensitivity of 96.5% (83 out of 86, 95% CI: 91.9%–100.0%) (Table 2).

**TABLE 2:** Diagnostic performance of DCE‐MRI BI‐RADS Category 4 NME using established TUS and CEUS downgrading criteria.

	Overall biopsy reduction		Benign biopsy reduction		Sensitivity	
	No. of lesions	Estimate (%)	No. of lesions	Estimate (%)	No. of lesions	Estimate (%)
TUS total	8/169	4.7 (1.8, 8.3)	6/83	7.2 (2.4, 13.3)	84/86	97.7 (94.2, 100.0)
TUS + CEUS total	39/169	23.1 (16.6, 29.6)	36/83	43.4 (32.5, 54.2)	83/86	96.5 (91.9, 100.0)
Ipsilateral symptoms						
Asymptomatic	19/58	32.8 (20.7, 44.8)	16/30	53.3 (36.7, 70.0)	25/28	89.3 (75.0, 100.0)
Symptomatic	20/111	18.0 (10.8, 25.2)	20/53	37.7 (24.5, 50.9)	58/58	100.0 (−)
*p* value		0.036		0.249		0.032
MRI BI‐RADS						
4a	33/101	32.7 (23.8, 41.6)	30/60	50.0 (38.3, 63.3)	38/41	92.7 (82.9, 100.0)
4b	5/41	12.2 (2.4, 21.9)	5/17	29.4 (11.8, 52.9)	24/24	100.0 (−)
4c	1/27	3.7 (0.0, 0.1)	1/6	16.7 (0.0, 50.0)	21/21	100.0 (−)
*p* value		0.001		0.171		0.322
FGT						
Almost entirely fat	3/7	42.9 (14.3, 85.7)	2/2	100.0 (−)	4/5	80.0 (40.0, 100.0)
Scattered fibroglandular tissue	12/66	18.2 (9.1, 27.3)	11/31	35.5 (19.4, 51.6)	34/35	97.1 (91.4, 100.0)
Heterogeneous fibroglandular tissue	21/79	26.6 (17.7, 36.7)	20/39	51.3 (35.9, 66.7)	39/40	97.5 (92.5, 100)
Extreme fibroglandular tissue	3/17	17.6 (0.0, 35.3)	3/11	27.3 (0.0, 54.5)	6/6	100.0 (−)
*p* value		0.346		0.135		0.329
Distribution of NME						
Focal	11/30	36.7 (20.0, 53.3)	10/16	62.5 (37.5, 87.5)	13/14	92.9 (78.6, 100.0)
Linear	2/7	28.6 (0.0, 57.1)	2/4	50.0 (0.0, 100.0)	3/3	100.0 (−)
Segmental	6/27	22.2 (7.4, 37.0)	5/12	41.7 (16.7, 66.7)	14/15	93.3 (80.0, 100.0)
Regional	17/84	20.2 (11.9, 29.7)	16/41	39.0 (24.4, 53.7)	42/43	97.7 (93.0,100.0)
Multiple regions	2/15	13.3 (0.0, 33.3)	2/8	25.0 (0.0, 50.0)	7/7	100.0 (−)
Diffuse	1/6	16.6 (0.0, 50.0)	1/2	50.0 (0.0, 100.0)	4/4	100.0 (−)
*p* value		0.488		0.562		0.625
Internal enhancement patterns						
Homogeneous	6/13	46.2 (23.1, 69.2)	6/10	60.0 (30.0, 90.0)	3/3	100.0 (−)
Heterogeneous	28/107	26.2 (18.7, 34.6)	25/48	52.1 (37.5, 66.6)	56/59	94.9 (88.1, 100.0)
Clumped	2/29	6.9 (0.0, 17.2)	2/8	25.0 (0.0, 50.0)	21/21	100.0 (−)
Clustered ring	3/20	15.0 (5.2, 36.1)	3/17	17.6 (0,0, 35.3)	3/3	100.0 (−)
*p* value		0.023		0.034		0.657
Lesion size on DCE‐MRI						
≤2 cm	21/52	40.4 (26.9, 53.8)	20/28	71.4 (53.6, 85.7)	23/24	95.8 (87.5, 100.0)
>2 cm	18/117	15.4 (9.40, 22.2)	16/55	29.1 (18.2, 41.8)	60/62	96.8 (91.9, 100.0)
*p* value		0.001		<0.001		0.831
Kinetic curve assessment						
I	16/46	34.8 (21.7, 47.8)	15/26	57.7 (38.5, 76.9)	19/20	95.0 (85.0, 100.0)
II	22/108	20.4 (13.0, 28.7)	20/53	37.7 (24.5, 50.9)	53/55	96.4 (90.0, 100.0)
III	1/15	6.7 (0.0, 20.0)	1/4	25.0 (0.0, 75.0)	11/11	100.0 (−)
*p* value		0.044		0.200		0.765

*Note:* TUS, targeted ultrasound; CEUS, contrast‐enhanced ultrasound; FGT, fibroglandular tissue.

Abbreviations: BI‐RADS, Breast Imaging Reporting and Data System; DCE‐MRI, dynamic contrast‐enhanced magnetic resonance imaging; NME, nonmass enhancement.

Of the 39 cases avoiding biopsy, 3 were false‐negative malignancies and 36 were benign (adenosis: 47.2%, *p* = 0.002 vs. non‐downgraded benign lesions). No significant differences in ER, PR, Ki‐67, grade, molecular subtype, T stage, or N stage were observed between false‐negative and true‐positive cases (Table 3).

**TABLE 3:** TUS + CEUS re‐evaluated the comparison of pathological features between cases requiring further biopsy and those without further biopsy.

	≤TUS + CEUS BI‐RADS 3 (%)	≥TUS + CEUS BI‐RADS 4 (%)	*p* value
Benign lesions (*n* = 83)	*n* = 36	*n* = 47	0.002
Adenosis	17 (47.2)	11 (23.4)	
Inflammation	4 (11.1)	23 (48.9)	
IDP/UDH/ADH/others^∗^	6/4/2/3 (16.7/11.1/5.6/8.3)	3/3/6/1 (6.4/6.4/12.8/2.1)	
Malignant lesions (*n* = 86)	*n* = 3	*n* = 83	
Pathological type			0.989
DCIS/with microinvasion/invasion	1/0/1 (33.3/0.0/33.3)	26/3/19 (31.3/3.6/22.9)	
IDC	1 (33.3)	26 (31.3)	
SPC/others^#^	—	4/5 (4.8/6.0)	
ER			0.869
Negative/positive	1/2 (33.3/66.7)	24/59 (28.9/71.1)	
PR			0.977
Negative/positive	1/2 (33.3/66.7)	27/56 (32.5/67.5)	
HER2			0.040
Negative/positive	0/3 (0.0/100.0)	56/27 (67.5/32.5)	
Ki67			0.549
≤ 14%/> 14%	1/2 (33.3/66.7)	30/53 (36.1/63.9)	
Grade			0.792
I or II/III	2/1 (33.3/33.3/33.3)	49/34 (59.0/41.0)	
Molecular typing			0.611
Luminal/HER2/TNBC	2/1/0 (66.7/33.3)	61/13/9 (73.5/15.7/10.8)	
pT‐stage			0.416
Tis/T1/T2–T4	1/2/0 (33.0/66.7/0.0)	26/27/30 (31.3/32.5/36.1)	
pN‐stage			0.146
N0/N1–N3	1/2 (33.0/66.7)	64/19 (77.1/22.9)	

*Note:* Others^∗^, including 3 fibroadenomas and 1 postoperative change. Others^#^, including 2 ILC with LCIS, 1 SPC with invasion, 1 invasive micropapillary carcinoma, and 1 mucous cancer with DCIS. TUS, targeted ultrasound. CEUS, contrast‐enhanced ultrasound. IDP, intraductal papilloma. HER2, human epidermal growth factor receptor 2.

Abbreviations: ADH, atypical ductal hyperplasia; BI‐RADS, Breast Imaging Reporting and Data System; DCIS, ductal carcinoma in situ; ER, estrogen receptor; IDC, invasive ductal carcinoma; ILC, invasive lobular carcinoma; LCIS, lobular carcinoma in situ; PR, progesterone receptor; SPC, solid papillary carcinoma; UDH, usual ductal hyperplasia.

Of three false‐negative cases, two were missed by CEUS, and one was downgraded. One missed case involved an occult breast lesion with enlarged axillary lymph nodes, diagnosed as low‐grade IDC (Luminal B) with 10% DCIS (MRI: 27 mm). The other was Paget’s disease with high‐grade HER2‐positive DCIS, showing 24 mm nonmass enhancement behind the left areola on MRI. The downgraded case was intermediate‐to‐high‐grade Luminal B DCIS with invasion, showing mild enhancement (8 × 12 mm) under the right areola on MRI, while CEUS indicated a slightly hyper‐enhanced lesion.

### 3.4. Stratified Performance Analysis of CEUS Application

Stratified analysis revealed higher MRI overestimation in asymptomatic cases, lesions ≤2 cm, homogeneous enhancement patterns, Type I kinetic curves, and MRI BI‐RADS 4a. Benign biopsy reduction was 53.3% (16/30) in asymptomatic versus 37.7% (20/53) in symptomatic cases; 71.4% (20/28) for lesions ≤2 cm versus 29.1% (16/55) for > 2 cm (*p* < 0.001); and 60.0% (6/10) for homogeneous enhancement (*p* = 0.034). Reduction rates by kinetic curve type were 57.7% (Type I), 37.7% (Type II), and 25.0% (Type III). For MRI BI‐RADS 4a, the reduction was 50.0% (30/60) versus 29.4% (4b) and 16.7% (4c) (Figures 3, 4, and 5). No intergroup differences in apparent diffusion coefficient (ADC) values were noted (Table 2, Table S2). Inter‐reader agreement for MRI features ranged from 0.814 to 0.942 (Table S1).

**FIGURE 3:**
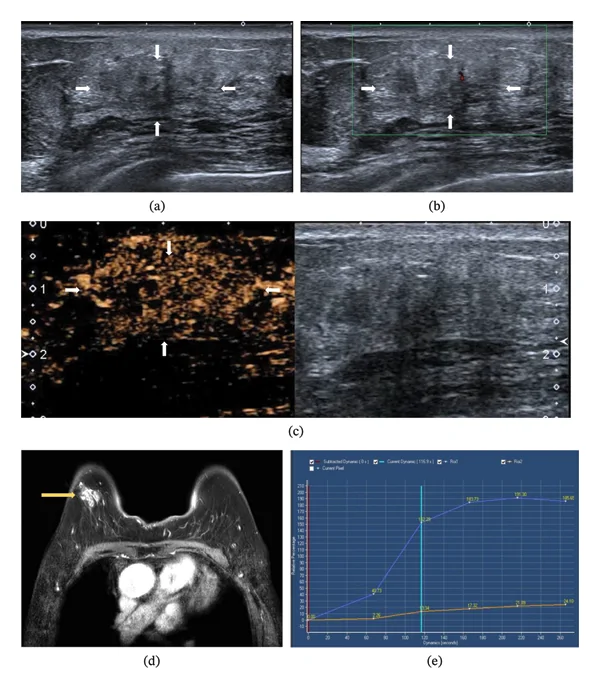
An 83‐year‐old female patient presented with a complaint of palpating a mass in the upper portion of the right breast accompanied by pain. Dynamic contrast‐enhanced magnetic resonance imaging (DCE‐MRI) revealed irregular punctate and patchy abnormal signal shadows in the upper portion of the right breast, with a diameter of approximately 1.5 × 1.2 cm. The time‐signal intensity curve was classified as Type II. The lesion was initially categorized as DCE‐MRI‐BI‐RADS Category 4a. Subsequently, targeted ultrasound and contrast‐enhanced ultrasound (CEUS) were performed for further evaluation. The lesion was downgraded to CEUS‐BI‐RADS Category 3 due to its iso‐enhancement during the arterial phase. The final histopathological diagnosis after surgical resection confirmed the lesion as adenosis.

**FIGURE 4:**
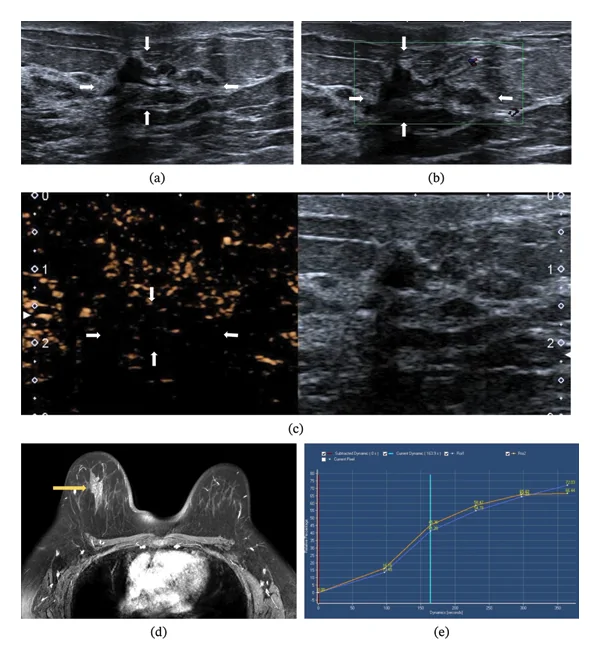
A 48‐year‐old female patient presented with a palpable mass. Dynamic contrast‐enhanced magnetic resonance imaging (DCE‐MRI) revealed nonmass‐like enhancement in the upper portion and the outer‐lower quadrant of the right breast, with a maximum diameter of less than 2 cm. The time‐signal intensity curve was classified as Type I, and the lesion was initially categorized as DCE‐MRI BI‐RADS Category 4a. Subsequent targeted ultrasound and contrast‐enhanced ultrasound (CEUS) examinations demonstrated iso‐enhancement, leading to a downgrade to CEUS‐BI‐RADS Category 3. Histopathological examination after surgical resection confirmed the lesion as adenosis.

**FIGURE 5:**
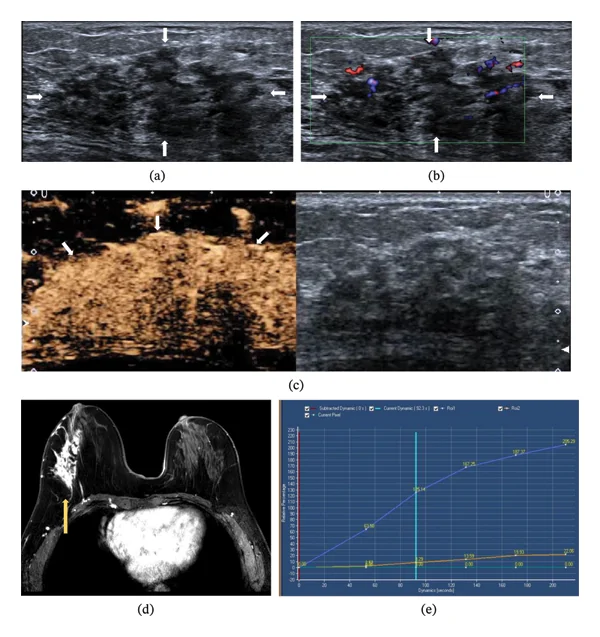
A 57‐year‐old female patient presented with a palpable mass in the outer portion of the right breast. Dynamic contrast‐enhanced magnetic resonance imaging (DCE‐MRI) revealed irregular patchy abnormal signal shadows in the outer upper and outer lower quadrants of the right breast, with the largest cross‐sectional dimensions measuring approximately 63 × 23 mm. The time‐signal intensity curve was classified as Type I, and the lesion was initially categorized as DCE‐MRI‐BI‐RADS Category 4a. Subsequent targeted ultrasound and contrast‐enhanced ultrasound (CEUS) examinations demonstrated earlier enhancement compared to the surrounding breast tissue, with heterogeneous enhancement. The enhanced area was larger than that observed on conventional ultrasound, with surrounding vascularization and contrast agent retention in the venous phase. Based on these findings, the lesion was upgraded to CEUS‐BI‐RADS Category 5. The final histopathological diagnosis after surgery was intermediate‐grade ductal carcinoma in situ with comedo‐type necrosis.

### 3.5. BI‐RADS Reclassification With TUS and CEUS

In benign NME reassessment, TUS combined CEUS downgraded 36 lesions to BI‐RADS 3 (*p* < 0.001). For malignant NME, CEUS upgraded 34 cases to BI‐RADS 4c/5 compared to MRI and 45 compared to TUS (*p* < 0.001) (Table 4). Of 24 DCIS or DCIS with invasion rated as MRI BI‐RADS 4a, 87.5% (21/24) were reclassified to 4b or higher with TUS + CEUS (Table S3). Inter‐reader agreement for BI‐RADS across modalities was good (kappa: 0.810–0.881, Table S1).

**TABLE 4:** The difference of BI‐RADS rating between DCE‐MRI, TUS, and CEUS for the BI‐RADS 4 category of NME.

BI‐RADS	Benign lesions (*n* = 83) (%)			Malignant lesions (*n* = 86) (%)		
	DCE‐MRI	TUS	TUS + CEUS	DCE‐MRI	TUS	TUS + CEUS
≤3	—	6 (7.2)	36 (43.4)	—	2 (2.3)	3 (3.5)
4a	60 (72.3)	67 (80.7)	22 (26.5)	41 (47.7)	43 (50.0)	3 (3.5)
4b	17 (20.5)	10 (12.0)	17 (20.5)	24 (27.9)	31 (36.0)	25 (29.0)
4c	6 (7.2)	—	8 (9.6)	21 (24.4)	10 (11.6)	33 (38.3)
5	—	—	—	—	—	22 (25.6)
*p* value	0.001^∗^	<0.001^#^	<0.001^§^	0.068^∗^	<0.001^#^	<0.001^§^

*Note:* TUS, targeted ultrasound. CEUS, contrast‐enhanced ultrasound.

Abbreviations: BI‐RADS, Breast Imaging Reporting and Data System; DCE‐MRI, dynamic contrast‐enhanced magnetic resonance imaging.

^∗^TUS versus DCE‐MRI.

^#^TUS + CEUS versus DCE‐MRI.

^§^TUS + CEUS versus TUS.

## 4. Discussion

This study demonstrates the value of combining TUS and CEUS for refining the diagnostic stratification of NME lesions initially classified as BI‐RADS 4 by DCE‐MRI. The combination reduced benign biopsy rates to 43.4% and overall biopsies by 23.1%, while maintaining high sensitivity (96.5%). CEUS further improved malignant NME detection, correctly reclassifying 87.5% of DCIS or DCIS with invasion from BI‐RADS 4a to 4b or higher, enhancing diagnostic confidence and biopsy targeting.

DCE‐MRI, despite high sensitivity, has variable positive predictive value for malignancy in NME (20.8%–93.8%), often overestimating malignant potential [18, 19]. Nonspecific enhancement patterns contribute to unnecessary biopsies, patient anxiety, and healthcare burden [5, 20, 21]. Subgroup analysis identified MRI overestimation factors: asymptomatic presentation, lesion size ≤2 cm, homogeneous enhancement, Type I kinetic curves, and BI‐RADS 4a classification, with biopsy reduction rates of 50%–71.4%. These results indicate that TUS combined with CEUS effectively refines risk stratification and reduces overtreatment, consistent with reported benign NME rates of 16.4%–75% [22].

TUS alone detects BI‐RADS 4 NME effectively but has limited ability to downgrade lesions, likely due to the lack of specific BI‐RADS criteria for NMLs, leading to conservative reporting [23]. CEUS overcomes this by providing microvascular information, allowing confident downgrading of MLs with well‐defined morphology and nonmass or adenosis lesions lacking enhancement or peripheral vessels. Malignant lesions, including IDC and DCIS, exhibit abundant vascularity, increased peripheral vessels, lesion enlargement, and prolonged contrast retention; venous‐phase hyperintensity further enhances detection of subtle microvascular structures, improving lesion characterization and reducing unnecessary biopsies [24, 25].

Three false‐negative cases illustrate CEUS limitations in anatomically complex regions. Two lesions were missed, and one was incorrectly downgraded, involving occult or subareolar lesions, low‐grade IDC, Paget disease with high‐grade DCIS, and intermediate‐to‐high‐grade DCIS with invasive foci. These cases highlight challenges in detecting subtle lesions and emphasize the need for multimodal imaging and careful clinical assessment.

Limitations include the single‐center design, limited sample size, and potential selection bias. Variations in equipment and operator experience may affect reproducibility. Elastography was not incorporated, which could have further improved diagnostic accuracy. CEUS did not provide additional findings for TUS‐invisible lesions, although prior studies suggest independent detection potential. Emerging methods such as deep learning and radiomics were not applied and warrant future investigation. Lesion localization relied on same‐day ultrasound‐guided skin marking rather than wire localization or clip placement, which may introduce greater spatial imprecision, particularly for small or deep lesions. The distance‐based localization strategy was originally validated in predominantly mass‐type lesions; although NME‐specific studies support its general applicability, our high TUS detection rate (97.0%) should be regarded as cohort‐ and operator‐specific, likely reflecting our exclusive focus on BI‐RADS 4 lesions, dedicated experienced radiologists, and a multilandmark localization protocol. These findings may not be fully generalizable to other settings. Finally, 22 lesions were excluded due to suboptimal image quality; their notably lower malignancy rate (18.2% vs. 50.9% in the final cohort) should be considered when interpreting the generalizability of the reported diagnostic performance.

In conclusion, TUS combined with CEUS effectively reassesses BI‐RADS 4 NME, reducing unnecessary biopsies while maintaining high sensitivity, particularly for small, homogeneously enhancing, or BI‐RADS 4a lesions. Careful application is required in complex regions, and multicenter studies with standardized protocols are needed to optimize diagnostic accuracy and safety.

## Author Contributions

Qinghua Niu and Ting Sun contributed equally to this work and are co‐first authors; An Chen and Fan Li contributed equally as co‐corresponding authors.

## Funding

This study was supported by the Shanghai Chest Hospital Technology Development Fund, Shanghai Jiao Tong University K. C. Wong Medical Fellowship Fund, Shanghai Municipal Education Commission, and Songjiang District Science and Technology Research Project (2023SJKWGG042).

## Conflicts of Interest

The authors declare no conflicts of interest.

## Supporting Information

Additional supporting information can be found online in the Supporting Information section.

## Supporting information


**Supporting Information** Table S1. Inter‐reader consistency for DCE‐MRI BI‐RADS 4 category NME lesions. Table S2. Comparison of ADC values in cases that avoided biopsy after TUS + CEUS reassessment versus cases that still required biopsy. Figure S1. Study flowchart illustrating prestudy criteria development and prospective per‐patient diagnostic workflow.

## Data Availability

The datasets generated and/or analyzed during the current study are available from the corresponding author on reasonable request.
