# Supplementary material for: Targeted Ultrasound With Contrast Enhancement for Reassessment of MRI‐Detected BI‐RADS 4 Nonmass Enhancement: Impact on Biopsy Reduction and DCIS Detection
Source: Breast J. 2026 Nov 5;2026:1932231. doi: 10.1155/tbj/1932231 (PMC13740486; doi:10.1155/tbj/1932231)
Supplement: Supplementary file 1 [file TBJ-2026-1932231-s001.docx]

**Supplementary Material**

| Table S1. Inter-reader Consistency for DCE-MRI BI-RADS 4 Category NME Lesions | | | |
| --- | --- | --- | --- |
| Factor | | Kappa value | 95%CI |
| DCE-MRI | |  |  |
|  | DCE-MRI BI-RADS | 0.814 | 0.733, 0.894 |
|  | FGT | 0.867 | 0.800, 0.934 |
|  | Distribution of NME | 0.898 | 0.843, 0.953 |
|  | Internal enhancement patterns | 0.903 | 0.854, 0.952 |
|  | Kinetic Curve assessment | 0.942 | 0.891, 0.993 |
| TUS | |  |  |
|  | TUS BI-RADS | 0.810 | 0.724, 0.896 |
| CEUS | |  |  |
|  | TUS+CEUS BI-RADS | 0.881 | 0.820, 0.942 |
|  | Enhanced time | 0.847 | 0.678, 1.000 |
|  | Enhanced Intensity at peak time | 0.914 | 0.840, 0.988 |
|  | Enhanced shape | 0.822 | 0.629, 1.000 |
|  | Emhanced margin | 0.830 | 0.646, 1.000 |
|  | Enhanced order | 0.911 | 0.846, 0.976 |
|  | Contrast agent distribution | 0.938 | 0.885, 0.990 |
|  | Increased surrounding vessel | 0.924 | 0.865, 0.983 |
|  | Perfusion defect | 0.957 | 0.908, 1.000 |
|  | Enhanced scope | 0.875 | 0.797, 0.950 |
|  | Contrast agent retention | 0.893 | 0.820, 0.966 |
| Note. DCE-MRI, Dynamic contrast-enhanced magnetic resonance imaging. BI-RADS, Breast Imaging Reporting and Data System. NME, Non-mass enhancement. FGT, Fibroglandular tissue. TUS, Targeted ultrasound. CEUS, Contrast-enhanced ultrasound. | | | |

| Table S2. Comparison of ADC values in cases that avoided biopsy after TUS+CEUS reassessment versus cases that still required biopsy | | | | | | | |
| --- | --- | --- | --- | --- | --- | --- | --- |
|  | | Total | | Benign | | Malignant | |
|  | | No biopsy needed | Biopsy needed | No biopsy needed | Biopsy needed | No biopsy needed | Biopsy needed |
| ADC value(×10^-3^mm^2^/sec) | |  |  |  |  |  |  |
|  | Maximum | 1.250(1.025, 1.485) | 1.200(0.940, 1.470) | 1.250(1.025, 1.485) | 1.220(0.980, 1.595) | 1.220(0.975, 1.590) | 1.190(0.905, 1.420) |
|  | *P* value |  | 0.423 |  | 0.964 |  | 0.945 |
|  | Minimum | 0.630(0.465, 0.970) | 0.600(0.370, 0.800) | 0.650(0.465, 0.970) | 0.640(0.520, 0.965) | 0.520(0.365, 0.980) | 0.550(0.285, 0.735) |
|  | *P* value |  | 0.250 |  | 0.836 |  | 0.871 |
|  | Mean | 0.880(0.750, 1.120) | 0.835(0.670, 1.060) | 0.865(0.750, 1.120) | 0.860(0.740, 1.285) | 0.880(0.655, 1.310) | 0.810(0.605, 0.975) |
|  | *P* value |  | 0.210 |  | 0.766 |  | 0.765 |
|  | SD of ADC | 0.120(0.100, 0.190) | 0.130(0.090, 0.180) | 0.125(0.095, 0.190) | 0.120(0.085, 0.155) | 0.120(0.110, 0.165) | 0.130(0.100, 0.190) |
|  | *P* value |  | 0.918 |  | 0.953 |  | 0.952 |
| Note. DCE-MRI, Dynamic contrast-enhanced magnetic resonance imaging. TUS, targeted ultrasound. CEUS, Contrast-enhanced ultrasound. ADC, apparent diffusion coefficient. | | | | | | | |

| Table S3. Comparative BI-RADS Ratings between DCE-MRI and TUS+CEUS in Malignant NME | | | | | | |
| --- | --- | --- | --- | --- | --- | --- |
| DCE-MRI BI-RADS | | TUS +CEUS BI-RADS | | | | |
|  |  | ≤3 | 4a | 4b | 4c | 5 |
| 4a(n=41) | | 3 | 2 | 16 | 12 | 8 |
|  | DCIS/with micro-invasion/invasion(n=24) | 2 | 1 | 13 | 5 | 3 |
|  | IDC(14) | 1 | 0 | 2 | 5 | 5 |
|  | Others^a^(n=3) | 0 | 1 | 1 | 1 | 0 |
| 4b(n=24) | |  |  |  |  |  |
|  | DCIS/with micro-invasion/invasion(n=17) | 0 | 0 | 4 | 11 | 2 |
|  | IDC(n=5) | 0 | 0 | 2 | 1 | 2 |
|  | SPC(n=2) | 0 | 1 | 1 | 0 | 0 |
| 4c(n=21) | |  |  |  |  |  |
|  | DCIS/with micro-invasion/invasion(n=8) | 0 | 0 | 1 | 3 | 4 |
|  | IDC(n=9) | 0 | 0 | 0 | 4 | 5 |
|  | Others^b^(n=4) | 0 | 0 | 1 | 2 | 1 |
| Note. Others^a^, including 1SPC, 1 Mucous cancer with DCIS and 1 micropapillary carcinoma. Others^b^, including 1SPC, 1 SPC with invasion and 2 ILC with LCIS.  DCE-MRI, Dynamic contrast-enhanced magnetic resonance imaging. TUS, targeted ultrasound. CEUS, Contrast-enhanced ultrasound. BI-RADS, Breast Imaging Reporting and Data System. DCIS, Ductal carcinoma In *situ*. IDC, Invasive ductal carcinoma. SPC, Solid papillary carcinoma. ILC, Invasive lobular carcinoma. LCIS, Lobular carcinoma in *situ*. | | | | | | |

Figure S1. Study flowchart illustrating pre-study criteria development and prospective per-patient diagnostic workflow.
